# Fiber-Modified Adenovirus for Central Nervous System Parkinson’s Disease Gene Therapy

**DOI:** 10.3390/v6083293

**Published:** 2014-08-21

**Authors:** Travis B. Lewis, Joel N. Glasgow, Ashley S. Harms, David G. Standaert, David T. Curiel

**Affiliations:** 1 Department of Cell Biology, The University of Alabama at Birmingham, Birmingham, AL 35294, USA; E-Mail: travis.lewis@uphs.upenn.edu; 2 Center for Neurodegeneration and Experimental Therapeutics, Department of Neurology, The University of Alabama at Birmingham, Birmingham, AL 35294, USA; E-Mails: anharms@uab.edu (A.S.H.); dstandaert@uab.edu (D.G.S.); 3 Department of Microbiology, University of Alabama at Birmingham, Birmingham, AL 35294, USA; E-Mail: jng@uab.edu; 4 Department of Radiation Oncology, School of Medicine, Washington University in St. Louis, St. Louis, MO 63108, USA

**Keywords:** gene therapy, adenovirus, brain, CNS, Parkinson disease

## Abstract

Gene-based therapies for neurological diseases continue to develop briskly. As disease mechanisms are elucidated, flexible gene delivery platforms incorporating transcriptional regulatory elements, therapeutic genes and targeted delivery are required for the safety and efficacy of these approaches. Adenovirus serotype 5 (Ad5)-based vectors can carry large genetic payloads to provide this flexibility, but do not transduce neuronal cells efficiently. To address this, we have developed a tropism-modified Ad5 vector with neuron-selective targeting properties for evaluation in models of Parkinson disease therapy. A panel of tropism-modified Ad5 vectors was screened for enhanced gene delivery in a neuroblastoma cell line model system. We used these observations to design and construct an unbiased Ad vector platform, consisting of an unmodified Ad5 and a tropism-modified Ad5 vector containing the fiber knob domain from canine Ad serotype 2 (Ad5-CGW-CK2). Delivery to the substantia nigra or striatum showed that this vector produced a neuronally-restricted pattern of gene expression. Many of the transduced neurons were from regions with afferent projections to the injection site, implicating that the vector binds the presynaptic terminal resulting in presynaptic transduction. We show that Ad5-CGW-CK2 can selectively transduce neurons in the brain and hypothesize that this modular platform is potentially adaptable to clinical use.

## 1. Introduction

There is great interest in developing gene therapy approaches to treat a wide range of central nervous system (CNS) disorders. In particular, gene therapy is considered a potentially valuable approach in the treatment of chronic neurodegenerative disorders, such as Parkinson disease, Alzheimer disease and amyotrophic lateral sclerosis [1,2]. In each of these disorders, genetic triggers, as well as impairments of specific metabolic pathways have been described, which could be amenable to gene therapy. There is also strong interest in generalized genetic interventions, such as induction of growth factor expression in the brain [3,4,5,6,7].

**Figure 1:**
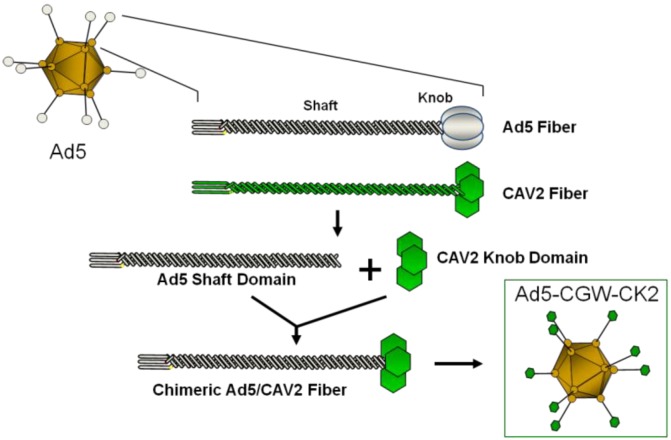
Schematic of fiber modification. Depiction of the Ad5 fiber replacement strategy. The fiber-modified Ad5-CGW-CK2 vector is structurally identical to Ad5, except for the knob domain of the cell-binding fiber protein. The Ad5 knob domain is genetically replaced by that of CAV2. Shaft and knob domains of Ad fiber proteins are shown.

Employing viral vectors in the brain poses special challenges. Currently used vector systems do not cross the blood-brain barrier and must be directly injected (requiring an invasive procedure, but providing physically targeted delivery; a notable exception is adeno-associated virus serotype 9 (AAV9)) [8,9,10]. Targeted, regional delivery is essential to minimize potential inflammatory reactions and to restrict therapeutic gene expression to the specific region of interest. Limiting transgene expression to neurons rather than glial cells may be more effective at interrupting intrinsic neurodegenerative processes. The majority of preclinical and clinical gene transfer studies in the CNS have utilized AAV-based vectors, which have an excellent immunogenicity profile and established clinical safety [11,12,13,14,15,16]. However, there are several limitations of the use of AAV. The most significant is the relatively small genome (4.7kB), which prohibits the inclusion of large transgenes and makes inclusion of disease- or tissue-specific transcriptional regulatory elements difficult or impossible [17]. 

Adenovirus serotype 5 (Ad5)-based vectors overcome the genetic packaging limitations of AAV (up to 36 kb in Ad *vs.* 4.7 kB in AAV) and allow for cell-selective transductional targeting of vector particles using bi-specific adaptor targeting molecules or by genetic modification of the Ad5 cellular attachment protein fiber (Figure 1) [18,19,20,21,22,23]. In previous studies, we observed that an Ad5 vector with native tropism provided limited gene transfer to the brain, with the majority of gene expression in glia rather than neurons. We showed that the basis for inefficient neuronal gene delivery was related to minimal neuronal expression of the coxsackie and adenovirus receptor (CAR), the primary Ad5 receptor. Further, transgenic expression of CAR in neurons led to greatly enhanced neuronal gene delivery, highlighting the requirement for tropism-modified CAR-independent Ad5 vectors for use in the CNS [24]. 

In this study, we screened a panel of tropism-modified Ad5-based vectors for transduction of neuroblastoma cell lines, which share some properties with human dopaminergic neurons. We selected a vector incorporating the canine adenovirus serotype 2 (CAV2) fiber C-terminal knob domain and constructed a new Ad5 vector with a reporter cassette encoding green fluorescent protein (Ad5-CGW-CK2) for evaluation in models of Parkinson disease therapy. Analysis of gene expression *in vivo* revealed that the tropism-modified Ad5-CGW-CK2 vector provides increased neuronal transduction and transgene expression compared to Ad5-CGW. This Ad-based platform may be of utility in next generation neuron-specific CNS gene therapy applications.

## 2. Results and Discussion

### 2.1. Gene Delivery in Dopaminergic Cell Lines Using Tropism-Modified Ad-Based Vectors

Two dopamine-producing human neuroblastoma cell lines were used to determine which structural modification to the Ad5 fiber protein would provide increased transduction. The two cell lines, SH-SY5Y and SK-N-BE (M17), while tumor-derived, retain some properties that are similar to the dopaminergic neurons that degenerate in Parkinson disease [25]. Additionally, these cells have been shown to be refractory to Ad5 vector transduction [26], also a feature of dopaminergic neurons *in vivo* directly related to the basis of this study.

We compared eleven tropism-modified Ad5 vectors that provide increased gene expression in a variety of CAR-deficient cells. These included: (1) fiber knob xenotyped vectors incorporating fiber knob domains from ovine Atadenovirus 7 [27], murine adenovirus serotype 1 [28], porcine adenovirus serotype 4 [29] and canine adenovirus serotypes 1 and 2 [23,30]; (2) a fiber pseudotyped vector, Ad5/3, that contains the Ad3 knob domain [31,32]; and (3) vectors with Ad5 fiber knob domains displaying artificial ligands, including poly-lysine (pK7), an integrin binding motif (RGD), pK7 and RGD ligands [33] and canine serotype 2 with poly-lysine, CK2-pK7 [34,35]. Gene delivery of each vector was compared to an unmodified Ad5 vector (Ad5Luc1) [23]. All vectors and the Ad5 control express firefly luciferase under control of the same cytomegalovirus (CMV) promoter. Gene delivery was quantified using luciferase-induced luminance. 

**Figure 2:**
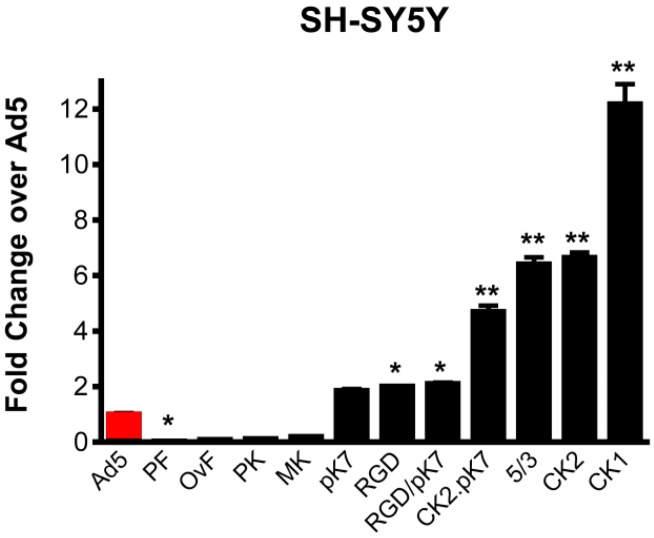
Ad5 vector gene transfer in dopaminergic cells. Luciferase reporter gene activity following transduction of SH-SY5Y human neuroblastoma cells with 10 viral particles (vp)/cell at 24 hours post-infection. Ad5-PF, porcine fiber and knob; Ad5-OvF, fiber protein from ovine Ad7; Ad5-PK, porcine Ad4 knob; Ad5-MK, murine Ad1 knob; Ad5-pK7 contains a cationic seven-lysine peptide incorporated into Ad5 knob; Ad5-RGD contains an arginine-glycine-aspartic acid peptide with integrin binding specificity incorporated into the Ad5 knob; Ad5-RGD/pK7 has both these modifications; Ad5-CK2.pK7 contains the CAV2 knob domain modified to include the poly-lysine motif; Ad5/3 is pseudotyped with the Ad3 knob domain; Ad5-CK2 has the CAV2 knob domain; Ad5-CK1 has the knob domain of CAV1. Statistical significance was assigned using one way ANOVA with Bonferroni selected comparison *post hoc* test. Error bars indicate the standard deviation. n = 4, * indicates *p* < 0.05, ** indicates *p* < 0.001 *vs.* Ad5.

Compared to unmodified Ad5Luc1, gene transfer to human SH-SY5Y cells was most significantly augmented by the canine knob-containing vectors, Ad5Luc1CK1 and Ad5Luc1CK2 (12-fold and seven-fold, respectively), while vectors containing the entire fiber protein (Ad5Luc1-PF) or knob domain (Ad5Luc1-PK) from porcine Ad4 displayed the lowest levels of gene delivery (Figure 2). Transgene expression levels in M17 cells were essentially the same as those in SH-SY5Y cells. 

### 2.2. Generation of an Unbiased Imaging Cassette and Incorporation into Fiber-Modified Adenovirus

We next examined the effect of tropism modification, particularly the incorporation of the CK2 knob domain, on neuronal gene delivery *in vivo.* Our selection of the CK2 modification from the original vector panel was based on the observed augmentation of gene delivery *in vitro*,as well as studies showing that canine adenovirus 2 (CAV2), which naturally includes the CK2 knob domain, provides highly neuron-selective transduction *in vivo* [36,37]. 

**Figure 3:**
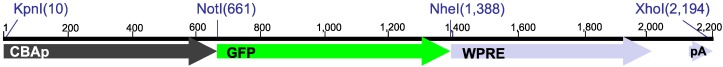
*In silico* design of a flexible expression cassette. KpnI, NotI, NheI and XhoI were designed to flank the pan-cellular cytomegalovirus (CMV)-enhanced chicken-β-actin (CAG) promoter, green fluorescent protein (GFP) for visualization of transduced cells and the woodchuck hepatitis virus posttranscriptional regulatory element (WPRE) to enhance mRNA stability. The cassette is designed to allow flexibility in replacing individual regions as a platform for additional applications. The unique, directional restriction sites that were incorporated utilize common buffers, allowing directional ligation of any insert. In addition, these restriction sites were chosen because they are not present in CMV, CAG or tyrosine hydroxylase (TH) promoters or other relevant transgenes, such as GFP, glial cell line-derived neurotrophic factor (GDNF), neurturin (NTN) and α-synuclein (ASYN).

Since our initial panel of tropism-modified Ad vectors relies on the quantitation of firefly luciferase activity to determine transduction efficiency and immunostaining for firefly luciferase *in vivo* proved unreliable, we developed additional vectors that encode green fluorescent protein (GFP), a more effective reporter for *in vivo* experiments (Figure 3). To allow a comparison with previous work using AAV vectors, we chose to utilize the pan-cellular cytomegalovirus-enhanced (CMV-enhanced) chicken-ß-actin (CBA) promoter (CAGp) to drive GFP expression, with mRNA stability enhanced by the woodchuck hepatitis virus post-transcriptional regulatory element (WPRE) [38,39]. Using the CAG promoter, GFP expression would be present in all cell types transduced by the Ad vector. Thus, we could assess differences in gene delivery between unmodified Ad5 and CK2-containing vectors to cellular subsets *in vivo.*

To maintain a flexible, modular expression cassette that would serve as a base for additional gene delivery strategies, we undertook in-depth *in silico* development of our expression cassette to maintain robust forward interoperability. This involved cloning unique restriction sites at locations 5’ to the promoter, between the promoter and transgene, between transgene and enhancer/poly-A elements and 3’ to the poly-A. Compatible sites share restriction buffer requirements, yet result in unique overhangs, allowing double restriction digests to excise either the promoter or transgene alone or the entire expression cassette, while allowing directional ligation of new elements back into the digested plasmid. We took additional steps to ensure that the restriction sites in the expression cassette are not present within the E1 shuttle plasmid, pShuttle or the Ad5 genome. Further, we verified that the restriction sites are not present in other DNA inserts relevant to *in vivo* models of Parkinson disease, including CMV, CAG and tyrosine hydroxylase (TH) promoters or in imaging, therapeutic and pathogenic transgenes, including GFP, glial cell line-derived neurotrophic factor (GDNF), neurturin (NTN) and α-synuclein (ASYN). Under these design constraints, we developed the pan-cellular expression cassette, CBAp-GFP-WPREpA (CGW), depicted schematically in Figure 3. 

**Figure 4:**
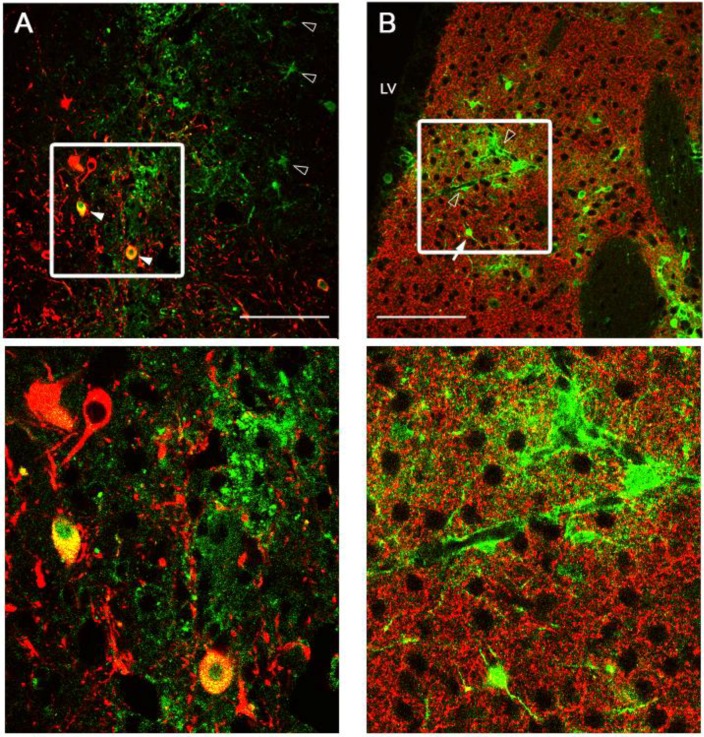
Pattern of Ad5-CBAp-GFP-WPREpA (CGW)-mediated GFP expression of in the substantia nigra (SN) and striatum (STR). Confocal immunohistochemical assessment of unmodified Ad5-CGW gene expression at the injection site two weeks after delivery to either SN (**A**) or STR (**B**) shows GFP transgene expression in a broad range of cell types, including cells with neuronal and astrocytic morphology, consistent with prior reports. Green = GFP expressing cells. Red = TH-positive neurons (A, TH-positive cell bodies in SN; B, TH-positive axon projections in STR). Solid arrowheads indicate GFP-expressing TH-positive neurons in the substantia nigra pars compacta (SNc). Open arrowheads indicate GFP-positive cells with astrocyte morphology. Solid arrows indicate GFP-positive cells with neuronal morphology in the STR. Inspection of presynaptic regions to the site of delivery did not show GFP transgene expression. LV, lateral ventricle. Bar = 100 µm.

**Figure 5:**
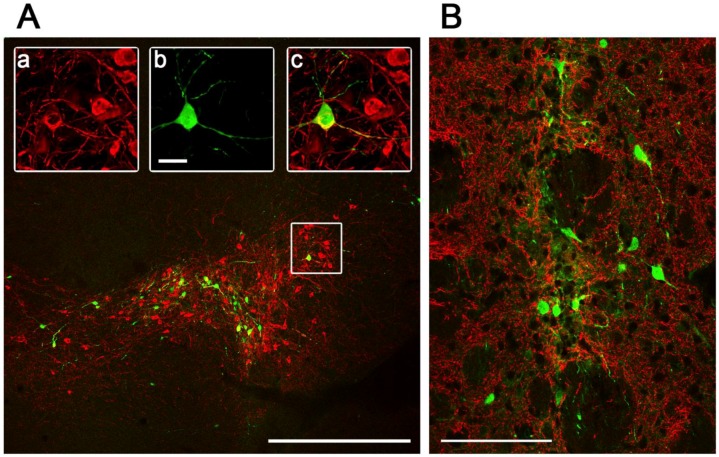
Pattern of Ad5-CGW-CK2-mediated GFP expression at the site of stereotactic delivery. Confocal immunohistochemical analysis of GFP expression by Ad5-CGW-CK2 stereotactically delivered to mouse SN (**A**) and STR (**B**) results in striking neuronal tropism. (**A**) Low-power image of the injected SN. Only neurons are positive for GFP expression. (**a**,**b**,**c**) Magnification of the region outlined in (**A**). A TH (red, a)/GFP (green, b) double-positive neuron is clearly visible surrounded by uninfected dopaminergic (DA) neurons (merge, c). (**B**) When Ad5-CGW-CK2 is delivered to the STR, GFP-expressing cells with neuronal morphology are observed. Despite thick (40 µm) sections and assessment of numerous slices around the injection site, no non-neuronal GFP expressing cells could be appreciated in the brains of animals receiving Ad5-CGW-CK2. Red = TH. Green = GFP. Bar in A = 500 μm. Bar in b (for a, b, c) = 2.5 μm. Bar in B = 100 μm.

### 2.3. Intracranial Delivery of Ad5-CGW-CK2 Provides Neuron-Specific Transgene Expression

Following production, purification and titering of Ad5-CGW and Ad5-CGW-CK2 vectors, we determined *in vivo* transduction profiles in the CNS via stereotactic delivery of 2 × 10^9^ vector particles (vp) to the SN or STR. One week post-injection, animals were sacrificed and immunohistochemical analysis of GFP expression was performed. Previous reports have described the native Ad5 transduction profile to include a broad range of CNS cell types centered at the site of injection. These include astrocytes, oligodendrocytes and neurons, with a preference for astrocytes [24]. We found a similar infection profile when Ad5-CGW is delivered to the SN, showing GFP expression in TH-positive neurons, as well as surrounding cells with astrocyte morphology (Figure 4A). Direct STR delivery of unmodified Ad5-CGW also displayed an infection profile with both neurons and non-neuronal cells expressing GFP (Figure 4B). Determinants of cell type were based on morphological analysis in non-TH positive neurons, and thus, it is possible that GFP positive cells are of an alternative cell type. Confocal analysis of coronal slices through the rostral-caudal axis showed that gene delivery was tightly restricted to the site of injection, with no appreciable GFP transgene expression at sites presynaptic to the injection site.

**Figure 6:**
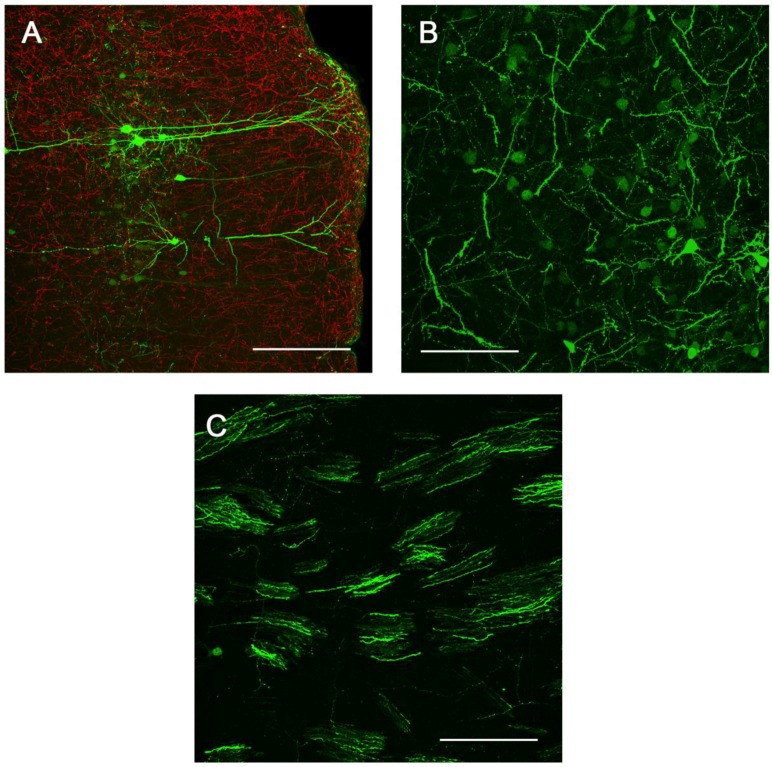
Sites of presynaptic GFP expression resulting from stereotactic delivery of Ad5-CGW-CK2 to SN. Regions with afferent projections to the SNc showed large numbers of neurons infected by Ad5-CGW-CK2. Regions presynaptic to the site of injection, including pyramidal neurons of the lateral cortex (**A**) and neurons of the globus pallidus (**B**), showed robust GFP expression. Additionally, GFP-positive fibers could be traced coursing through the STR in bundled myelinated fibers (**C**, correlating to the human internal capsule). All images are flattened confocal z-stacks through the regions indicated. Green = GFP. Red = TH. Bar in A = 200 µm, Bar in B and C = 100 µm.

**Figure 7:**
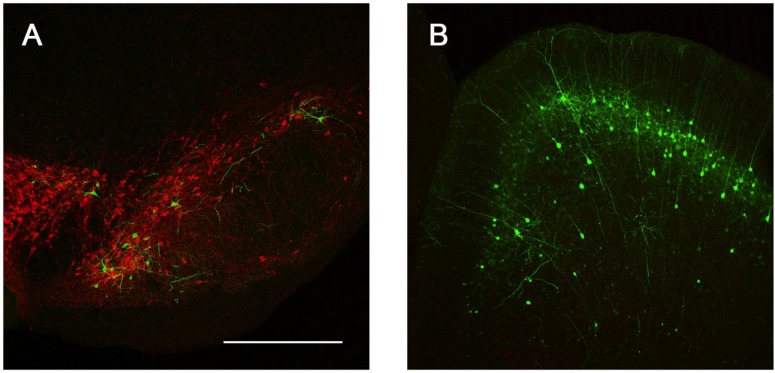
Sites of presynaptic GFP expression resulting from stereotactic delivery of Ad5-CGW-CK2 to the STR. Following injection into the STR, robust transgene expression was noted in distal regions with afferent projections to this motor control region. These include the SNc (**A**) and pyramidal neurons of the dorsal cortex (**B**). B is a flattened confocal z-stack through the dorsal cortex. Green = GFP. Red = TH. Bar is 500 µm for both panels.

We next determined the *in vivo* transduction profile of Ad5-CGW-CK2. When delivered to the SN, we found striking neuronal tropism, with many neurons double-labeled for GFP and the dopaminergic marker tyrosine hydroxylase (TH). Based on morphology, there was little evidence of non-neuronal staining. Determinants of cell type were based on morphological analysis in non-TH positive neurons, and thus, it is possible that GFP-positive cells are of an alternative cell type (Figure 5). A second, and unanticipated difference between Ad5-CGW and Ad5-CGW-CK2 gene expression was the transduction pattern observed in neurons distant from the site of injection. While Ad5-CGW transduced local neuronal and non-neuronal cells, Ad5-CGW-CK2 led to the infection of a large number of neurons in distal regions, particularly those known to have axons projecting into the injection site. In SN-injected animals, we observed numerous pyramidal neurons of the lateral cortex strongly positive for GFP (Figure 6A, correlating to the presynaptic motor cortex by atlas coordinates), as well as robust GFP expression in neurons of the globus pallidus (GP, Figure 6B) and coursing through the STR in bundled myelinated fibers (Figure 6C, correlating to the human internal capsule). Following striatal injection, there were small numbers of transduced neurons near the injection site with the morphology of medium spiny neurons (Figure 5B), but there was robust GFP expression in regions with afferent projections to the STR, including the SN (particularly SNc, Figure 7A), as well as the dorsal cortex (Figure 7B, correlating by atlas coordinates to the presynaptic somatosensory cortex).

## 3. Experimental Section

### 3.1. In Vitro Fiber-Modified Vector Panel Screen

SH-SY5Y or M17 cells were plated on 24-well culture plates in DMEM/F12 media supplemented with 10% fetal bovine serum (FBS) for 80% confluence the following day and incubated at 37 °C. Twenty four hours later, media was changed to low-serum infection media (1% FBS) for one hour prior to infection. Each fiber-modified vector (expressing firefly luciferase via the CMV promoter) was delivered with a multiplicity of infection (MOI) of 10 vp/cell, in triplicate. Infection proceeded for one hour at 37 °C before virus-containing infection media was gently removed and replaced with standard growth media containing 10% FBS. Twenty-four hours post-infection, growth medium was removed and cells were gently rinsed with PBS. Luciferase activity was assessed according to the manufacturer (Promega, Madison, WI, USA). Briefly, cells were lysed with reporter lysis buffer, scraped, and transferred to individual microcentrifuge tubes. Cell debris was pelleted, and 20 μL of supernatant were added to 100 μL of luciferase assay reagent. Luminometer readings were normalized to the relative light units (RLU) resulting from unmodified Ad5 infection.

### 3.2. Construction of the Transgene Expression Cassette by Polymerase Chain Assembly

Polymerase chain assembly (PCA) was used to produce the CGW transgene expression cassette [40]. The CAGp/NotI/GFP segment of our cassette utilized the primer sequence: CCGGGGGCGGTGCCCCGCGG
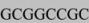
ATGGTGAGCAAGGGC where the underlined sequence is complimentary to the 3’ end of the CAGp, the gray highlighted sequence is a *de novo* NotI restriction site and the plain-text sequence is complimentary to the 5’ end of GFP. To complete the cassette, we designed a 5’CAGp primer incorporating a KpnI restriction site (AAAA
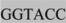
ATCGAGGTGAGCCCCACGTT), a 3’ GFP/NheI/5’ WPRE primer (GAGCTGTACAAGTAA
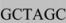
AATCAACCTCTGGATTACAA) and a 5’ WPRE/XhoI primer (GGAATTTTTTGTGTCTCTCA
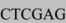
AAAA). Using the appropriate primers and the source template for CAGp, GFP or WPRE-polyA, 3 PCR reactions were performed to derive the three overlapping segments containing the appropriate restriction sites (GC-Rich PCR system, Roche Applied Science, Mannheim, Germany). Using the three overlapping segments, we performed an extension step to create a template of the full-length CGW cassette and, finally, amplified this cassette using the distal 5’ and 3’ primers, producing the complete KpnI/CAGp/NotI/GFP/NheI/WPRE-pA/XhoI construct.

### 3.3. Generation of Recombinant Adenovirus Vectors

The CGW transgene expression cassette described above was inserted into the multiple cloning site of the E1 shuttle plasmid pShuttle to generate pShuttle-CGW. The recombinant Ad5 genome containing the wild-type Ad5 fiber gene was derived by homologous recombination in *E. coli* BJ5183 with PacI-linearized rescue plasmid pAdEasy1 and PmeI-linearized pShuttle-CGW. The recombinant Ad5 genome containing the chimeric Ad5 fiber with the CK2 knob domain was derived similarly, but required a derivative of pAdEasy1, wherein the Ad5 fiber was replaced by a chimeric fiber incorporating the knob domain of CAV2. The resultant Ad5 genomic clones were sequenced and analyzed by PCR to confirm the presence of GFP and Ad5 hexon genes. Validated genomic clones were transformed into DH5α *E. coli* and upscaled.

Genomic clones were linearized with PacI and transfected into HEK293 cells using polyethylenimine (PEI). All vectors were propagated on HEK293 cells and purified by double equilibrium centrifugation through CsCl gradients followed by dialysis to remove CsCl. The viral particle (vp) concentration was determined by absorbance at 260 nm by the method of Maizel *et al.* by using a conversion factor of 1.1 × 10^12^ vp/absorbance unit [41]. 

### 3.4. Stereotactic Vector Delivery

To determine the transduction profile of Ad5 vectors *in vivo*, we stereotactically delivered 2 × 10^9^ viral particles (vp) of Ad5-CGW-CK2 or Ad5-CGW in a 2-μL volume to either the SN or STR using five animals per group. Mice were anesthetized with inhaled isoflurane and immobilized on a stereotactic frame. The skull was exposed, and bregma was located. The coordinates for right SN injection were: anterior-posterior, −3.1 mm from bregma, medio-lateral, −1.2 from midline and dorso-ventral, −3.8 from the dura. The coordinates for right STR injection were: anterior-posterior, +0.8 mm from bregma, medio-lateral, −1.7 from midline and dorso-ventral, −3.4 from the dura. Vector was injected at a flow rate of 0.25 µL/minute over eight minutes, followed by two minutes for diffusion before the syringe was slowly retracted. The incision was sealed, and animals were allowed to recover on a warming pad.

### 3.5. Immunohistochemistry

One week post-injection, animals were deeply anesthetized and transcardially perfused with 4% paraformaldehyde in phosphate buffered saline (PBS). Brains were removed and post-fixed for two hours at room temperature before cryopreservation by impregnation with 30% sucrose in PBS for 48 hours at 4 °C. Tissue was flash frozen in isopentane on dry ice, and 40-μm sections were cut using a sliding microtome and collected as free-floating tissue in PBS:glycerol, 1:1. For GFP and TH staining, floating sections were blocked with 6% normal goat serum for 60 minutes followed by incubation with mouse anti-GFP 1:10,000 (MAB3580, Millipore, Billerica, MA, USA) and rabbit anti-TH 1:2000 (P40101-0, PelFreez Biologicals, Rogers, AR, USA) for 24 hours at 25 °C. Tissue was then washed in PBS, followed by incubation in a 1:5000 dilution of alexa-488 conjugated goat anti-mouse (Molecular Probes, Carlsbad, CA, USA) and a 1:500 dilution of alexa-555 conjugated goat anti-rabbit (Jackson Immunoresearch, West Grove, PA, USA) secondary antibodies. Slides were then coverslipped with Vectashield mounting medium (Vector Labs, Burlingame, CA, USA).

### 3.6. Imaging

Confocal images were captured using a Leica TCS-SP5 laser scanning confocal microscope. The images were processed using the Leica LAS AF 2.6.3 (Leica Microsystems, Wetzlar Germany) and exported as Tiff files and post-processed using Adobe Photoshop CS3. 

## 4. Conclusions

The primary goal of this study was to develop and evaluate a tropism-modified adenoviral vector for use in CNS gene therapy, particularly for utility in Parkinson disease. We began with *in vitro* analysis of multiple fiber-modified Ad vectors, showing that these modifications can elicit a broad range of transduction efficiencies (covering a 100-fold range) in two neuroblastoma cell lines. Based on the efficacy of the Ad5Luc1-CK2 vector *in vitro*, as well as prior *in vivo* studies using CAV2-based vectors [35,36], we produced and evaluated a new CK2 knob-modified Ad vector (Ad5-CGW-CK2). This vector exhibited strong neural tropism, with negligible transduction of non-neuronal cells based on morphological assessment. Surprisingly, Ad5-CGW-CK2 also exhibited a pattern of neuronal transgene expression that included neurons with afferent projections to the site of vector delivery. These data led us to propose that the binding target for this vector is present on presynaptic terminals, facilitating a presynaptic pattern of transduction that warrants further study. 

When screening our panel of tropism-modified Ad5-based vectors *in vitro*, we measured firefly luciferase activity to identify tropism-modified vectors that provided markedly increased gene expression compared to standard Ad5 (Figure 2). While fiber-mediated tropism modification is by far the most likely cause of the increased gene expression observed *in vitro*, we cannot rule out that other factors (such as more efficient intracellular trafficking of internalized vector particles) may also contribute to increased gene expression. Indeed, the fiber protein is a major determinant in intracellular trafficking and the fate of internalized virions [42,43,44]. Numerous studies have shown that many Ad5 vectors containing structurally-modified Ad5 fibers or alternate serotype fibers exhibit intracellular trafficking defects resulting in reduced and/or delayed gene expression compared to native Ad5 [42,43,44,45]. A similar property would only serve to underestimate the relative transduction efficiency of the fiber-modified vectors used in this study. More important to our primary findings regarding neuronal gene delivery *in vivo* is that native Ad5 and canine adenovirus serotype 2 (CAV2), the virus from which the fiber knob domain of Ad5-CGW-CK2 was derived, were observed to have virtually identical kinetics of internalization, endosomal escape, nuclear localization and replication [46]. 

The Ad5-CGW and Ad5-CGW-CK2 vectors are isogenic, except for the fiber knob domain. This eliminates variables that could otherwise confound side-by-side comparison, such as variations in transgene expression linked to non-identical promoters and variations in vector production. To accomplish this, we included identical pan-cellular expression cassettes and manipulated only the C-terminal knob domain of the fiber protein. Our results confirm that the CK2 fiber modification alone is sufficient to restrict vector transduction to neurons, and the receptor moiety appears to be present both on the postsynaptic neuron (resulting in the expected local transgene expression) and on the presynaptic terminal (resulting in a presynaptic pattern of expression). We have yet to identify the CK2 target on these cells, although other groups have identified potential binding partners for canine adenovirus serotype-2 (CAV-2) that naturally uses the CK2 knob domain [47,48,49]. Further studies into the neuronal target(s) utilized by the CK2 knob for binding and transducing cells will be necessary to shed light on this process. It is not yet clear from our studies whether this additional presynaptic pattern of GFP distribution in Ad5-CGW-CK2 infected neurons is the result of a novel mechanism of vector particle transport secondary to a capsid-mediated process, the presence of a novel presynaptic binding partner or a combination of these; the studies do not elucidate whether the vector, the uncoated DNA or the translated transgene are involved in this novel pattern. Since the only difference between Ad5-CGW and Ad5-CGW-CK2 is the modification of the external knob domain (Figure 1), the additional presynaptic pattern of expression is most likely the result of the knob domain binding a partner on the presynaptic terminal that Ad5-CGW does not recognize. Thus, for example, when Ad5-CGW-CK2 is infused into the STR, not only is the particle taken up by local cell bodies, but axon terminals are additionally targeted, resulting in the above described presynaptic pattern of expression. Of note, Castle *et al.* have shown that transport of AAV9 particles within the neuron is a complex and active process involving the anterograde motor kinesin-2 and Rab7-mediated trafficking within the late endosome/lysosome for retrograde transport; processes that are almost certainly distinct from the mechanism of transgene distribution throughout the cell [50]. Microfluidics studies in primary neuron cultures would help elucidate this mechanism. Additionally, our studies suggest that the CK1 modification may also have potential utility in neuronal transduction, as indicated by a higher gene delivery than the CK2 modification *in vitro*. While we chose to focus on the CK2 fiber modification, it would be of interest to compare the CNS gene delivery of other vectors capable of a high transduction of neuroblastoma cell lines (Ad5/3 and CK1).

The long-term goal of this work is to develop a neuron-targeted gene therapy vector platform that can be used in human clinical trials. While our *in vivo* data showing neuron-restricted transgene expression by Ad5-CGW-CK2 is in a murine model, primary screening was performed in human tumor-derived cell lines. It would be informative to repeat these *in vitro* studies on more stringent substrates, such as human and mouse primary neuron cultures. It will be important to assess the targeting of Ad5-CGW-CK2 in non-human primates and post-mortem human tissue to validate this model before evaluation in humans. In addition, we limited our early studies to brain regions affected by Parkinson disease, and further study in other brain areas to assess gene delivery profiles will clearly be of interest.

A remaining question is the mechanism by which Ad5-CGW-CK2 provides neuron-restricted transgene expression. We have narrowed the likely step to cell attachment and/or internalization, as the structure of the fiber knob domain that mediates this process is the only independent variable. It will be important to identify the neuronal receptor(s) of the CK2 vector in order to better understand this mechanism prior to advancing the vector towards clinical use.

As described previously, an important strength of Ad5-based vectors is the large payload capacity allowing incorporation of tissue-specific transcriptional control elements to regulate the location, quantity and duration of transgene expression. Our data shows that fiber modification alone is sufficient for transductional targeting, however we have not addressed the use of physiologically relevant transcriptional control elements. It is likely that clinical gene therapy strategies, which combine transductional and disease-relevant transcriptional controls to fine-tune gene expression, will provide the highest therapeutic efficacy, while also maximizing clinical safety.

In addition to the precise control of transgene expression, the duration of transgene expression is a major consideration. Treatment for long-duration degenerative processes will likely require long-duration transgene expression. In this regard, prior studies show that high-capacity adenoviral vectors (HCAd or “gutless” Ad vectors) can maintain transgene expression in the CNS for over a year [37,51]. In general, the CNS appears to be immunologically protected against developing a vector-neutralizing immunologic response following repeated exposures [52,53]. Once a more detailed understanding of the mechanism of Ad5-CGW-CK2 binding and infection is appreciated, an important next step will be the transition of this transductional approach to HCAd vectors.
